# Should Infants Be Separated from Mothers with COVID-19? First, Do No Harm

**DOI:** 10.1089/bfm.2020.29153.ams

**Published:** 2020-05-08

**Authors:** Alison Stuebe

The COVID-19 pandemic has affected every facet of life, beginning with the first hours and days after birth. In an effort to “bend the curve,” some have argued that the prudent course is to isolate infants from mothers with suspected or confirmed COVID-19 in order to reduce the risk of transmission from mother to baby.

Data are limited, and recommendations for the first days after birth differ. The World Health Organization (WHO)^1^ recommends that infants and mothers with suspected or confirmed COVID-19 “should be enabled to remain together and practice skin-to-skin contact, kangaroo care and to remain together and to practice rooming in throughout the day and night.” Breastfeeding is strongly recommended, given its known lifelong importance for maternal and child health. Mothers are encouraged to wash their hands, wear a mask if they have a cough, and routinely disinfect surfaces that they have touched.

The United States Centers for Disease Control and Prevention (CDC)^2^ advises that facilities “consider temporarily separating the mother from her infant” until the mother is no longer considered contagious. During separation, the CDC recommends that women express breast milk, to be fed to the newborn by a healthy caregiver. If rooming in is preferred by the mother or unavoidable due to facility limitations, steps to reduce risk are described. The CDC further suggests that “the risks and benefits of temporary separation should be discussed by the healthcare team,” but does not elaborate.

New data are emerging daily, and by the time this commentary is published, the landscape may have changed dramatically. But as of late March 2020, what might be the benefits and risks of temporary separation?

The benefit of separation is that it minimizes the risk of transmission of SARS-CoV-2 from mother to infant during the hospital stay. However, if the goal is the health and well-being of mother and child in the months following birth, there are additional considerations.

(1) *Separation may not prevent infection.* A study published in late March reported that 3 of 33 infants born in Wuhan, China, to mothers with COVID-19 tested positive for SARS-CoV-2; the infants were born by cesarean and managed with strict isolation precautions.^3^ Even if separation prevents infection during the maternity stay, it does not address exposure after the infant is discharged. Especially in the context of social distancing and travel restrictions, few families have the resources to isolate the infant at home, and it is highly plausible that other household members may be infected. Hospital isolation may therefore delay, but not prevent, infant infection.
(2) *Interruption of skin-to-skin care disrupts newborn physiology*. Infants who are separated from their mothers have higher heart rates and respiratory rates and lower glucose levels than infants who are skin-to-skin.^4^ This holds true even for infants who are placed in incubators. In a randomized controlled trial for 1200- to 2199-gram newborns, among infants who were skin-to-skin, 17% of infants experienced instability, based on objective parameters, compared to 92% of the infants in conventional incubators.^5^ In a subsequent study among term infants placed skin-to-skin versus alone in a crib, separation increased stress activity by 176%.^6^ As noted by the Royal College of Obstetricians and Gynecologists, “routine precautionary separation of a mother and a healthy baby should not be undertaken lightly, given the potential detrimental effects on feeding and bonding.”^7^ Isolation is a significant stressor for newborn infants; for those infants already infected with SARS-CoV-2, isolation could worsen the disease course.
(3) *Separation stresses mothers*. When mothers held their preterm infants skin-to-skin in the neonatal intensive care unit, their heart rate, salivary cortisol level, and stress scores decreased.^8^ Separating mothers from their infants, especially in the context of being diagnosed with a pandemic disease, has the potential to cause significant suffering, and the associated physiologic stress could worsen the mother's disease course.
(4) *Separation interferes with provision of maternal milk to the infant, disrupting innate and specific immune protection.* Breastfeeding is a baby's first vaccine, and skin-to-skin care is important for colonization of the infant microbiome. Antibodies specific to maternal antigen exposure begin to appear in milk within 7 days,^9^ protecting the infant from infection. Furthermore, human milk contains multiple oligosaccharides and innate immune factors that mitigate the impact of viral infections.
(5) *Early separation disrupts breastfeeding, and not breastfeeding increases the risk of infant hospitalization for pneumonia.* Early separation decreases breastfeeding duration compared to keeping mothers and infants together. And when infants are not breastfed, they have 3.6 times the risk of being hospitalized for pneumonia compared to infants who are exclusively breastfed for ≥4 months.^10^ Separating mother and baby immediately after birth may make the infant more vulnerable to severe respiratory infections, including COVID-19, in the first year of life.
(6) *Separate isolation doubles the burden on the health system*. Separately isolating mother and infant requires twice the resources: two hospital rooms, two provider teams, and two sets of personal protective equipment (PPE) each time a provider enters or leaves the room. In the context of hospital overcrowding and dangerous shortages of PPE, this is deeply problematic.

In the United States, technology and clinical science have long been “normal,” whereas skin-to-skin contact and rooming in defy the reductionism of Western medicine. In contrast, officials at the WHO remember the lessons of the human immunodeficiency virus epidemic, where recommendations to substitute formula for breastfeeding had devastating consequences in low-income countries.^11^ At the time of writing, we have no evidence to show that early separation improves outcomes. As we navigate the COVID-19 pandemic, I am hopeful that we can center mothers and babies and remember to first do no harm.
